# *N*-(3-Methyl­benzo­yl)benzene­sulfonamide

**DOI:** 10.1107/S1600536812013931

**Published:** 2012-04-06

**Authors:** P. A. Suchetan, Sabine Foro, B. Thimme Gowda

**Affiliations:** a Department of Chemistry, Mangalore University, Mangalagangotri 574 199, Mangalore, India; b Institute of Materials Science, Darmstadt University of Technology, Petersenstrasse 23, D-64287 Darmstadt, Germany

## Abstract

The asymmetric unit of the title compound, C_14_H_13_NO_3_S, contains three independent mol­ecules in which the dihedral angles between the sulfonyl and benzoyl benzene rings are 83.3 (2), 84.4 (2) and 87.6 (2)°. In the crystal, mol­ecules are linked into chains running along the *a* axis *via* N—H⋯O hydrogen bonds.

## Related literature

For our studies on the effects of substituents on the structures and other aspects of *N*-(ar­yl)-amides, see: Gowda *et al.* (2000 ▶, 2007 ▶), on *N*-(substitutedbenzo­yl)-aryl­sulfonamides, see: Gowda *et al.* (2009 ▶), on *N*-chloro­aryl­amides, see: Jyothi & Gowda (2004 ▶) and on *N*-bromo­aryl­sulfonamides, see: Usha & Gowda (2006 ▶).
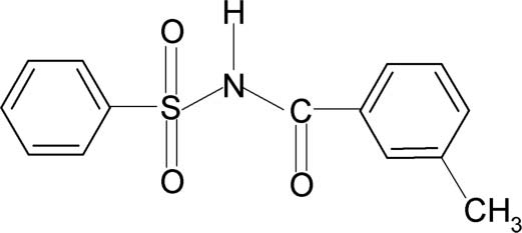


## Experimental

### 

#### Crystal data

C_14_H_13_NO_3_S
*M* *_r_* = 275.31
Monoclinic, 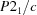

*a* = 11.6028 (8) Å
*b* = 35.100 (3) Å
*c* = 10.4886 (8) Å
β = 100.920 (7)°
*V* = 4194.2 (6) Å^3^
*Z* = 12
Mo *K*α radiation
μ = 0.23 mm^−1^
*T* = 293 K
0.46 × 0.30 × 0.04 mm

#### Data collection

Oxford Diffraction Xcalibur diffractometer with a Sapphire CCD detector
Absorption correction: multi-scan (*CrysAlis RED*; Oxford Diffraction, 2009 ▶) *T*_min_ = 0.900, *T*_max_ = 0.991
15537 measured reflections
7317 independent reflections
4440 reflections with *I* > 2σ(*I*)
*R*_int_ = 0.045

#### Refinement

*R*[*F* ^2^ > 2σ(*F* ^2^)] = 0.097
*wR*(*F* ^2^) = 0.146
*S* = 1.29
7317 reflections
526 parameters
3 restraints
H atoms treated by a mixture of independent and constrained refinement
Δρ_max_ = 0.30 e Å^−3^
Δρ_min_ = −0.33 e Å^−3^

### 

Data collection: *CrysAlis CCD* (Oxford Diffraction, 2009 ▶); cell refinement: *CrysAlis RED* (Oxford Diffraction, 2009 ▶); data reduction: *CrysAlis RED*; program(s) used to solve structure: *SHELXS97* (Sheldrick, 2008 ▶); program(s) used to refine structure: *SHELXL97* (Sheldrick, 2008 ▶); molecular graphics: *PLATON* (Spek, 2009 ▶); software used to prepare material for publication: *SHELXL97*.

## Supplementary Material

Crystal structure: contains datablock(s) I, global. DOI: 10.1107/S1600536812013931/bt5866sup1.cif

Structure factors: contains datablock(s) I. DOI: 10.1107/S1600536812013931/bt5866Isup2.hkl

Supplementary material file. DOI: 10.1107/S1600536812013931/bt5866Isup3.cml

Additional supplementary materials:  crystallographic information; 3D view; checkCIF report

## Figures and Tables

**Table 1:** Hydrogen-bond geometry (Å, °)

*D*—H⋯*A*	*D*—H	H⋯*A*	*D*⋯*A*	*D*—H⋯*A*
N1—H1*N*⋯O7^i^	0.85 (2)	2.04 (2)	2.864 (5)	164 (5)
N2—H2*N*⋯O2^ii^	0.86 (2)	2.15 (3)	2.920 (5)	150 (5)
N3—H3*N*⋯O6	0.85 (2)	2.05 (2)	2.891 (5)	172 (5)

Symmetry codes: (i) 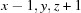; (ii) 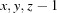.

**Fig. 1.**
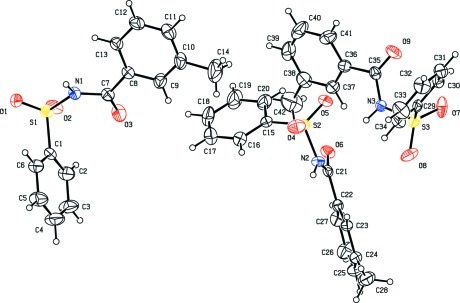
Molecular structure of the title compound, showing the atom- labelling scheme. Displacement ellipsoids are drawn at the 50% probability level.

**Fig. 2.**
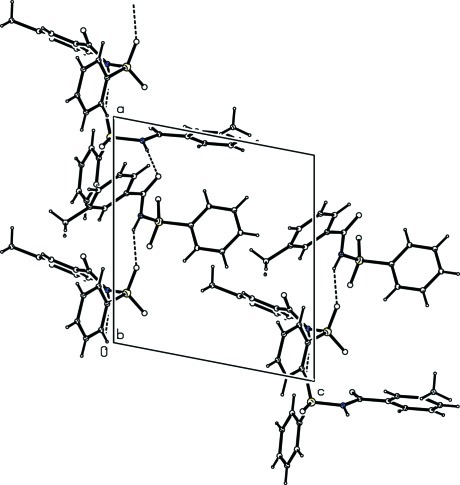
Molecular packing in the title compound. Hydrogen bonds are shown as dashed lines.

## supplementary crystallographic information

### Comment

Diaryl acylsulfonamides are known as potent antitumor agents against a broad spectrum of human tumor xenografts (colon, lung, breast, ovary and prostate) in nude mice. As part of our studies on the substituent effects on the structures and other aspects of *N*-(aryl)-amides (Gowda *et al.*, 2000, 2007), *N*-(substitutedbenzoyl)-arylsulfonamides (Gowda *et al.*, 2009), *N*-chloroarylsulfonamides (Jyothi & Gowda, 2004) and *N*-bromoarylsulfonamides (Usha & Gowda, 2006), in the present work, the crystal structure of *N*-(3-methylbenzoyl)benzenesulfonamide has been determined (Fig.1).

The conformation of the N—H bond in the C—SO_2_—NH—C(O) segment is *anti* to the C=O bond (Fig.1), similar to that observed in *N*-(3-chlorobenzoyl)benzenesulfonamide (I)(Gowda *et al.*, 2009).

In the title compound, the dihedral angles between the sulfonyl benzene rings and the —SO_2_—NH—C—O segments are 76.6 (2)°, 82.2 (2)° and 78.4 (2)°, compared to the value of 79.6 (1)° in (I).

The dihedral angles between the sulfonyl and the benzoyl benzene rings are 83.3 (2)°, 84.4 (2)° and 87.6 (2)°, compared to the value of 89.3 (1)° in (I).

The packing of molecules linked by of N—H···O hydrogen bonds (Table 1) is shown in Fig. 2.

### Experimental

The title compound was prepared by refluxing a mixture of 3-methylbenzoic acid, benzene sulfonamide and phosphorous oxy chloride for 5 h on a water bath. The resultant mixture was cooled and poured into ice cold water. The solid obtained was filtered, washed thoroughly with water and then dissolved in sodium bicarbonate solution. The compound was later reprecipitated by acidifying the filtered solution with dilute HCl. The filtered and dried solid was recrystallized to the constant melting point.

Plate like colourless single crystals of the title compound used in X-ray diffraction studies were obtained from a slow evaporation of the solvent from its toluene solution at room temperature.

### Refinement

The coordinates of the H atoms bonded to N were refined with the to N—H distance restrained to 0.86 (2) %A. The other H atoms were positioned with idealized geometry using a riding model with C—H distances of 0.93 Å (C-aromatic) and 0.96 Å (*C*-methyl).

All H atoms were refined with isotropic displacement parameters were set at 1.2 *U*_eq_(C_aromatic_, N) and 1.5 *U*_eq_(C_methyl_).

The (1 1 1) reflection is probably affected by the beamstop and was omitted from the refinement.

### Crystal data

**Table:**

C_14_H_13_NO_3_S	*F*(000) = 1728
*M**_r_* = 275.31	*D*_x_ = 1.308 Mg m^−^^3^
Monoclinic, *P*2_1_/*c*	Mo *K*α radiation, λ = 0.71073 Å
Hall symbol: -P 2ybc	Cell parameters from 3245 reflections
*a* = 11.6028 (8) Å	θ = 2.6–27.9°
*b* = 35.100 (3) Å	µ = 0.23 mm^−^^1^
*c* = 10.4886 (8) Å	*T* = 293 K
β = 100.920 (7)°	Plate, colourless
*V* = 4194.2 (6) Å^3^	0.46 × 0.30 × 0.04 mm
*Z* = 12	

### Data collection

**Table:**

Oxford Diffraction Xcalibur diffractometer with a Sapphire CCD detector	7317 independent reflections
Radiation source: fine-focus sealed tube	4440 reflections with *I* > 2σ(*I*)
Graphite monochromator	*R*_int_ = 0.045
Rotation method data acquisition using ω scans	θ_max_ = 25.0°, θ_min_ = 2.6°
Absorption correction: multi-scan (*CrysAlis RED*; Oxford Diffraction, 2009)	*h* = −8→13
*T*_min_ = 0.900, *T*_max_ = 0.991	*k* = −41→28
15537 measured reflections	*l* = −12→12

### Refinement

**Table:**

Refinement on *F*^2^	Primary atom site location: structure-invariant direct methods
Least-squares matrix: full	Secondary atom site location: difference Fourier map
*R*[*F*^2^ > 2σ(*F*^2^)] = 0.097	Hydrogen site location: inferred from neighbouring sites
*wR*(*F*^2^) = 0.146	H atoms treated by a mixture of independent and constrained refinement
*S* = 1.29	*w* = 1/[σ^2^(*F*_o_^2^) + (0.*P*)^2^ + 7.5834*P*] where *P* = (*F*_o_^2^ + 2*F*_c_^2^)/3
7317 reflections	(Δ/σ)_max_ < 0.001
526 parameters	Δρ_max_ = 0.30 e Å^−^^3^
3 restraints	Δρ_min_ = −0.33 e Å^−^^3^

### Special details

**Table:**

Experimental. CrysAlis RED (Oxford Diffraction, 2009) Empirical absorption correction using spherical harmonics, implemented in SCALE3 ABSPACK scaling algorithm.
Geometry. All e.s.d.'s (except the e.s.d. in the dihedral angle between two l.s. planes) are estimated using the full covariance matrix. The cell e.s.d.'s are taken into account individually in the estimation of e.s.d.'s in distances, angles and torsion angles; correlations between e.s.d.'s in cell parameters are only used when they are defined by crystal symmetry. An approximate (isotropic) treatment of cell e.s.d.'s is used for estimating e.s.d.'s involving l.s. planes.
Refinement. Refinement of *F*^2^ against ALL reflections. The weighted *R*-factor *wR* and goodness of fit *S* are based on *F*^2^, conventional *R*-factors *R* are based on *F*, with *F* set to zero for negative *F*^2^. The threshold expression of *F*^2^ > σ(*F*^2^) is used only for calculating *R*-factors(gt) *etc*. and is not relevant to the choice of reflections for refinement. *R*-factors based on *F*^2^ are statistically about twice as large as those based on *F*, and *R*- factors based on ALL data will be even larger.

### Fractional atomic coordinates and isotropic or equivalent isotropic displacement parameters (Å^2^)

**Table:**

	*x*	*y*	*z*	*U*_iso_*/*U*_eq_	
C1	0.1661 (4)	0.20368 (14)	0.9680 (5)	0.0404 (13)	
C2	0.2356 (5)	0.23047 (17)	0.9252 (6)	0.0666 (18)	
H2	0.3169	0.2286	0.9466	0.080*	
C3	0.1831 (7)	0.2603 (2)	0.8495 (7)	0.089 (2)	
H3	0.2294	0.2785	0.8192	0.107*	
C4	0.0632 (7)	0.2631 (2)	0.8189 (7)	0.088 (2)	
H4	0.0282	0.2833	0.7686	0.105*	
C5	−0.0049 (6)	0.2361 (2)	0.8625 (7)	0.078 (2)	
H5	−0.0863	0.2380	0.8410	0.094*	
C6	0.0452 (5)	0.20613 (16)	0.9377 (6)	0.0565 (16)	
H6	−0.0014	0.1879	0.9675	0.068*	
C7	0.2962 (5)	0.12424 (15)	0.8764 (5)	0.0456 (14)	
C8	0.2903 (4)	0.08623 (14)	0.8128 (5)	0.0398 (13)	
C9	0.3140 (5)	0.08350 (17)	0.6894 (6)	0.0600 (17)	
H9	0.3306	0.1055	0.6470	0.072*	
C10	0.3136 (6)	0.04891 (19)	0.6271 (6)	0.0654 (18)	
C11	0.2922 (5)	0.01670 (18)	0.6924 (7)	0.0638 (18)	
H11	0.2939	−0.0069	0.6528	0.077*	
C12	0.2686 (5)	0.01875 (17)	0.8150 (7)	0.0646 (18)	
H12	0.2539	−0.0034	0.8579	0.078*	
C13	0.2665 (5)	0.05358 (15)	0.8749 (6)	0.0518 (15)	
H13	0.2488	0.0550	0.9576	0.062*	
C14	0.3375 (9)	0.0467 (2)	0.4911 (7)	0.142 (4)	
H14A	0.4206	0.0455	0.4943	0.170*	
H14B	0.3059	0.0688	0.4432	0.170*	
H14C	0.3011	0.0242	0.4492	0.170*	
N1	0.2269 (4)	0.12842 (11)	0.9691 (4)	0.0408 (11)	
H1N	0.172 (3)	0.1133 (11)	0.979 (5)	0.049*	
O1	0.1566 (3)	0.15579 (10)	1.1538 (3)	0.0563 (10)	
O2	0.3507 (3)	0.17572 (10)	1.1137 (4)	0.0588 (11)	
O3	0.3556 (4)	0.15030 (11)	0.8490 (4)	0.0723 (13)	
S1	0.23040 (12)	0.16583 (4)	1.06531 (14)	0.0439 (4)	
C15	0.5844 (5)	0.12158 (15)	0.3849 (5)	0.0477 (14)	
C16	0.4896 (5)	0.14058 (17)	0.4163 (6)	0.0617 (17)	
H16	0.4226	0.1448	0.3536	0.074*	
C17	0.4954 (7)	0.1532 (2)	0.5411 (8)	0.083 (2)	
H17	0.4322	0.1662	0.5634	0.099*	
C18	0.5945 (8)	0.1468 (2)	0.6327 (7)	0.095 (3)	
H18	0.5977	0.1552	0.7174	0.113*	
C19	0.6884 (7)	0.1283 (2)	0.6011 (7)	0.099 (3)	
H19	0.7554	0.1242	0.6640	0.118*	
C20	0.6843 (6)	0.11550 (19)	0.4757 (6)	0.074 (2)	
H20	0.7482	0.1030	0.4533	0.089*	
C21	0.6831 (4)	0.16510 (14)	0.1471 (5)	0.0372 (12)	
C22	0.6719 (4)	0.19969 (14)	0.0646 (5)	0.0373 (12)	
C23	0.5861 (4)	0.20471 (14)	−0.0453 (5)	0.0399 (13)	
H23	0.5313	0.1855	−0.0707	0.048*	
C24	0.5803 (5)	0.23784 (17)	−0.1181 (5)	0.0515 (15)	
C25	0.6612 (6)	0.26632 (17)	−0.0762 (7)	0.0692 (19)	
H25	0.6570	0.2891	−0.1223	0.083*	
C26	0.7470 (7)	0.26166 (19)	0.0314 (7)	0.082 (2)	
H26	0.8012	0.2810	0.0574	0.098*	
C27	0.7531 (5)	0.22833 (17)	0.1012 (6)	0.0609 (17)	
H27	0.8123	0.2250	0.1736	0.073*	
C28	0.4903 (6)	0.2429 (2)	−0.2401 (6)	0.083 (2)	
H28A	0.4304	0.2238	−0.2437	0.100*	
H28B	0.5273	0.2403	−0.3142	0.100*	
H28C	0.4556	0.2677	−0.2405	0.100*	
N2	0.5845 (4)	0.14267 (12)	0.1351 (4)	0.0414 (11)	
H2N	0.516 (2)	0.1500 (13)	0.099 (4)	0.050*	
O4	0.4570 (3)	0.09120 (10)	0.1793 (4)	0.0667 (12)	
O5	0.6724 (4)	0.08030 (10)	0.2222 (4)	0.0646 (12)	
O6	0.7731 (3)	0.15704 (10)	0.2228 (3)	0.0512 (10)	
S2	0.57382 (13)	0.10423 (4)	0.22610 (14)	0.0492 (4)	
C29	0.8038 (4)	0.06031 (14)	−0.0737 (4)	0.0378 (13)	
C30	0.8412 (5)	0.02571 (15)	−0.1099 (5)	0.0462 (14)	
H30	0.9209	0.0204	−0.1005	0.055*	
C31	0.7585 (6)	−0.00106 (17)	−0.1605 (5)	0.0592 (16)	
H31	0.7826	−0.0246	−0.1867	0.071*	
C32	0.6408 (6)	0.00656 (19)	−0.1728 (6)	0.0678 (19)	
H32	0.5857	−0.0118	−0.2066	0.081*	
C33	0.6045 (5)	0.04131 (19)	−0.1353 (6)	0.0711 (19)	
H33	0.5247	0.0463	−0.1432	0.085*	
C34	0.6850 (5)	0.06871 (17)	−0.0862 (5)	0.0548 (16)	
H34	0.6607	0.0924	−0.0618	0.066*	
C35	0.9608 (4)	0.06864 (16)	0.2280 (5)	0.0430 (14)	
C36	0.9756 (4)	0.07833 (17)	0.3685 (5)	0.0479 (14)	
C37	1.0054 (5)	0.11455 (18)	0.4134 (5)	0.0580 (17)	
H37	1.0161	0.1336	0.3550	0.070*	
C38	1.0198 (6)	0.1230 (2)	0.5453 (6)	0.076 (2)	
C39	1.0025 (6)	0.0938 (3)	0.6276 (7)	0.090 (3)	
H39	1.0115	0.0988	0.7160	0.108*	
C40	0.9728 (6)	0.0580 (3)	0.5849 (7)	0.092 (3)	
H40	0.9613	0.0391	0.6433	0.110*	
C41	0.9598 (5)	0.04983 (19)	0.4545 (7)	0.0698 (19)	
H41	0.9405	0.0253	0.4245	0.084*	
C42	1.0549 (8)	0.1623 (2)	0.5946 (7)	0.138 (4)	
H42A	1.0151	0.1809	0.5347	0.166*	
H42B	1.1382	0.1653	0.6025	0.166*	
H42C	1.0337	0.1659	0.6779	0.166*	
N3	0.9179 (4)	0.09775 (12)	0.1442 (4)	0.0393 (10)	
H3N	0.881 (4)	0.1165 (10)	0.167 (5)	0.047*	
O7	1.0178 (3)	0.08427 (12)	−0.0409 (4)	0.0700 (13)	
O8	0.8578 (4)	0.13197 (11)	−0.0578 (3)	0.0686 (12)	
O9	0.9847 (4)	0.03767 (11)	0.1896 (4)	0.0700 (12)	
S3	0.90607 (13)	0.09578 (4)	−0.01381 (13)	0.0471 (4)	

### Atomic displacement parameters (Å^2^)

**Table:**

	*U*^11^	*U*^22^	*U*^33^	*U*^12^	*U*^13^	*U*^23^
C1	0.038 (3)	0.037 (3)	0.045 (3)	0.006 (3)	0.006 (3)	−0.006 (3)
C2	0.050 (4)	0.057 (4)	0.094 (5)	0.002 (3)	0.019 (4)	0.014 (4)
C3	0.078 (6)	0.070 (5)	0.123 (7)	0.004 (4)	0.030 (5)	0.046 (5)
C4	0.084 (6)	0.074 (5)	0.101 (6)	0.019 (4)	0.008 (5)	0.037 (5)
C5	0.058 (4)	0.079 (5)	0.092 (6)	0.016 (4)	−0.002 (4)	0.017 (4)
C6	0.051 (4)	0.051 (4)	0.066 (4)	0.001 (3)	0.008 (3)	0.007 (3)
C7	0.042 (3)	0.040 (3)	0.055 (4)	−0.005 (3)	0.011 (3)	−0.003 (3)
C8	0.030 (3)	0.037 (3)	0.052 (4)	−0.007 (2)	0.007 (3)	−0.006 (3)
C9	0.074 (4)	0.054 (4)	0.058 (4)	−0.011 (3)	0.028 (3)	−0.002 (3)
C10	0.082 (5)	0.066 (5)	0.052 (4)	−0.009 (4)	0.023 (4)	−0.022 (4)
C11	0.067 (4)	0.048 (4)	0.077 (5)	−0.009 (3)	0.014 (4)	−0.024 (4)
C12	0.077 (5)	0.039 (4)	0.082 (5)	−0.003 (3)	0.024 (4)	−0.004 (4)
C13	0.057 (4)	0.044 (4)	0.057 (4)	0.000 (3)	0.018 (3)	−0.003 (3)
C14	0.239 (11)	0.116 (7)	0.092 (7)	−0.016 (8)	0.085 (7)	−0.037 (6)
N1	0.038 (3)	0.034 (3)	0.052 (3)	−0.010 (2)	0.014 (2)	−0.008 (2)
O1	0.078 (3)	0.050 (2)	0.045 (2)	0.006 (2)	0.021 (2)	−0.0014 (19)
O2	0.046 (2)	0.048 (2)	0.072 (3)	0.0000 (19)	−0.015 (2)	−0.012 (2)
O3	0.078 (3)	0.047 (2)	0.104 (4)	−0.026 (2)	0.049 (3)	−0.018 (2)
S1	0.0458 (9)	0.0375 (8)	0.0454 (9)	0.0031 (7)	0.0012 (7)	−0.0060 (7)
C15	0.060 (4)	0.037 (3)	0.051 (4)	0.006 (3)	0.023 (3)	0.010 (3)
C16	0.068 (4)	0.063 (4)	0.057 (4)	0.006 (3)	0.020 (3)	0.006 (3)
C17	0.093 (6)	0.085 (5)	0.082 (6)	0.011 (5)	0.045 (5)	−0.002 (5)
C18	0.132 (7)	0.110 (7)	0.052 (5)	0.005 (6)	0.045 (5)	−0.006 (5)
C19	0.105 (6)	0.143 (8)	0.046 (5)	0.024 (6)	0.010 (4)	0.006 (5)
C20	0.075 (5)	0.099 (6)	0.047 (4)	0.021 (4)	0.009 (4)	0.014 (4)
C21	0.043 (3)	0.036 (3)	0.034 (3)	0.004 (3)	0.010 (3)	−0.008 (3)
C22	0.034 (3)	0.039 (3)	0.040 (3)	−0.003 (2)	0.010 (2)	−0.004 (3)
C23	0.046 (3)	0.031 (3)	0.045 (3)	−0.002 (3)	0.013 (3)	0.001 (3)
C24	0.054 (4)	0.053 (4)	0.051 (4)	0.013 (3)	0.020 (3)	0.013 (3)
C25	0.097 (6)	0.041 (4)	0.078 (5)	0.000 (4)	0.038 (4)	0.015 (4)
C26	0.095 (6)	0.064 (5)	0.089 (6)	−0.037 (4)	0.023 (5)	0.002 (4)
C27	0.054 (4)	0.064 (4)	0.063 (4)	−0.017 (3)	0.007 (3)	−0.006 (4)
C28	0.081 (5)	0.092 (5)	0.079 (5)	0.017 (4)	0.023 (4)	0.037 (4)
N2	0.041 (3)	0.037 (3)	0.044 (3)	0.004 (2)	0.001 (2)	0.007 (2)
O4	0.070 (3)	0.048 (2)	0.078 (3)	−0.019 (2)	0.004 (2)	0.006 (2)
O5	0.086 (3)	0.044 (2)	0.066 (3)	0.026 (2)	0.020 (2)	0.006 (2)
O6	0.043 (2)	0.056 (2)	0.048 (2)	0.0163 (19)	−0.0081 (19)	−0.0036 (19)
S2	0.0638 (10)	0.0343 (8)	0.0493 (9)	0.0036 (8)	0.0100 (7)	0.0068 (7)
C29	0.046 (3)	0.044 (3)	0.025 (3)	−0.002 (3)	0.010 (2)	−0.004 (2)
C30	0.047 (3)	0.054 (4)	0.038 (3)	0.002 (3)	0.008 (3)	−0.008 (3)
C31	0.078 (5)	0.050 (4)	0.049 (4)	0.000 (3)	0.008 (3)	−0.012 (3)
C32	0.065 (5)	0.063 (5)	0.071 (5)	−0.022 (4)	0.001 (4)	−0.017 (4)
C33	0.043 (4)	0.078 (5)	0.088 (5)	−0.001 (4)	0.000 (3)	−0.015 (4)
C34	0.054 (4)	0.052 (4)	0.057 (4)	0.004 (3)	0.006 (3)	−0.009 (3)
C35	0.035 (3)	0.050 (4)	0.043 (4)	0.005 (3)	0.006 (3)	−0.006 (3)
C36	0.039 (3)	0.057 (4)	0.046 (4)	0.012 (3)	0.002 (3)	0.007 (3)
C37	0.070 (4)	0.066 (4)	0.033 (3)	0.016 (3)	−0.003 (3)	0.004 (3)
C38	0.084 (5)	0.091 (5)	0.044 (4)	0.034 (4)	−0.013 (4)	−0.012 (4)
C39	0.075 (5)	0.157 (8)	0.032 (4)	0.044 (6)	−0.004 (4)	0.008 (5)
C40	0.081 (6)	0.142 (8)	0.049 (5)	0.012 (6)	0.006 (4)	0.038 (5)
C41	0.063 (4)	0.076 (5)	0.066 (5)	0.004 (4)	0.002 (4)	0.023 (4)
C42	0.194 (10)	0.126 (8)	0.072 (6)	0.045 (7)	−0.030 (6)	−0.040 (6)
N3	0.045 (3)	0.041 (3)	0.031 (2)	0.006 (2)	0.005 (2)	−0.002 (2)
O7	0.054 (3)	0.102 (3)	0.064 (3)	−0.032 (2)	0.036 (2)	−0.029 (2)
O8	0.109 (3)	0.049 (3)	0.042 (2)	−0.019 (2)	−0.001 (2)	0.012 (2)
O9	0.081 (3)	0.054 (3)	0.071 (3)	0.029 (2)	0.004 (2)	−0.009 (2)
S3	0.0568 (9)	0.0533 (9)	0.0331 (8)	−0.0165 (8)	0.0134 (7)	−0.0054 (7)

### Geometric parameters (Å, º)

**Table:**

C1—C2	1.369 (7)	C23—C24	1.386 (7)
C1—C6	1.381 (7)	C23—H23	0.9300
C1—S1	1.754 (5)	C24—C25	1.384 (8)
C2—C3	1.383 (8)	C24—C28	1.501 (8)
C2—H2	0.9300	C25—C26	1.366 (9)
C3—C4	1.371 (9)	C25—H25	0.9300
C3—H3	0.9300	C26—C27	1.374 (8)
C4—C5	1.367 (9)	C26—H26	0.9300
C4—H4	0.9300	C27—H27	0.9300
C5—C6	1.375 (8)	C28—H28A	0.9600
C5—H5	0.9300	C28—H28B	0.9600
C6—H6	0.9300	C28—H28C	0.9600
C7—O3	1.212 (6)	N2—S2	1.671 (4)
C7—N1	1.381 (6)	N2—H2N	0.857 (19)
C7—C8	1.487 (7)	O4—S2	1.426 (4)
C8—C13	1.372 (7)	O5—S2	1.426 (4)
C8—C9	1.377 (7)	C29—C30	1.367 (7)
C9—C10	1.378 (8)	C29—C34	1.391 (7)
C9—H9	0.9300	C29—S3	1.753 (5)
C10—C11	1.369 (8)	C30—C31	1.376 (7)
C10—C14	1.505 (8)	C30—H30	0.9300
C11—C12	1.367 (8)	C31—C32	1.374 (8)
C11—H11	0.9300	C31—H31	0.9300
C12—C13	1.377 (7)	C32—C33	1.372 (8)
C12—H12	0.9300	C32—H32	0.9300
C13—H13	0.9300	C33—C34	1.371 (7)
C14—H14A	0.9600	C33—H33	0.9300
C14—H14B	0.9600	C34—H34	0.9300
C14—H14C	0.9600	C35—O9	1.209 (6)
N1—S1	1.652 (4)	C35—N3	1.378 (6)
N1—H1N	0.849 (19)	C35—C36	1.491 (7)
O1—S1	1.422 (4)	C36—C37	1.377 (7)
O2—S1	1.435 (3)	C36—C41	1.382 (7)
C15—C20	1.370 (7)	C37—C38	1.394 (8)
C15—C16	1.379 (7)	C37—H37	0.9300
C15—S2	1.756 (6)	C38—C39	1.381 (10)
C16—C17	1.372 (8)	C38—C42	1.502 (9)
C16—H16	0.9300	C39—C40	1.355 (10)
C17—C18	1.370 (9)	C39—H39	0.9300
C17—H17	0.9300	C40—C41	1.378 (9)
C18—C19	1.363 (9)	C40—H40	0.9300
C18—H18	0.9300	C41—H41	0.9300
C19—C20	1.382 (8)	C42—H42A	0.9600
C19—H19	0.9300	C42—H42B	0.9600
C20—H20	0.9300	C42—H42C	0.9600
C21—O6	1.220 (5)	N3—S3	1.638 (4)
C21—N2	1.375 (6)	N3—H3N	0.846 (19)
C21—C22	1.482 (7)	O7—S3	1.437 (4)
C22—C27	1.382 (7)	O8—S3	1.429 (4)
C22—C23	1.384 (6)		
			
C2—C1—C6	121.2 (5)	C25—C24—C23	118.2 (5)
C2—C1—S1	119.9 (4)	C25—C24—C28	120.4 (6)
C6—C1—S1	118.9 (4)	C23—C24—C28	121.4 (6)
C1—C2—C3	119.0 (6)	C26—C25—C24	121.3 (6)
C1—C2—H2	120.5	C26—C25—H25	119.4
C3—C2—H2	120.5	C24—C25—H25	119.4
C4—C3—C2	120.4 (6)	C25—C26—C27	119.9 (6)
C4—C3—H3	119.8	C25—C26—H26	120.1
C2—C3—H3	119.8	C27—C26—H26	120.1
C5—C4—C3	119.8 (6)	C26—C27—C22	120.5 (6)
C5—C4—H4	120.1	C26—C27—H27	119.7
C3—C4—H4	120.1	C22—C27—H27	119.7
C4—C5—C6	120.9 (6)	C24—C28—H28A	109.5
C4—C5—H5	119.5	C24—C28—H28B	109.5
C6—C5—H5	119.5	H28A—C28—H28B	109.5
C5—C6—C1	118.7 (6)	C24—C28—H28C	109.5
C5—C6—H6	120.7	H28A—C28—H28C	109.5
C1—C6—H6	120.7	H28B—C28—H28C	109.5
O3—C7—N1	121.3 (5)	C21—N2—S2	124.1 (3)
O3—C7—C8	123.4 (5)	C21—N2—H2N	124 (3)
N1—C7—C8	115.3 (5)	S2—N2—H2N	109 (3)
C13—C8—C9	118.8 (5)	O5—S2—O4	121.1 (2)
C13—C8—C7	122.2 (5)	O5—S2—N2	108.2 (2)
C9—C8—C7	118.9 (5)	O4—S2—N2	103.4 (2)
C8—C9—C10	121.6 (6)	O5—S2—C15	108.6 (3)
C8—C9—H9	119.2	O4—S2—C15	109.2 (3)
C10—C9—H9	119.2	N2—S2—C15	105.2 (2)
C11—C10—C9	118.3 (6)	C30—C29—C34	121.5 (5)
C11—C10—C14	120.9 (6)	C30—C29—S3	120.1 (4)
C9—C10—C14	120.7 (6)	C34—C29—S3	118.3 (4)
C12—C11—C10	121.0 (6)	C29—C30—C31	118.6 (5)
C12—C11—H11	119.5	C29—C30—H30	120.7
C10—C11—H11	119.5	C31—C30—H30	120.7
C11—C12—C13	120.0 (6)	C32—C31—C30	120.8 (6)
C11—C12—H12	120.0	C32—C31—H31	119.6
C13—C12—H12	120.0	C30—C31—H31	119.6
C8—C13—C12	120.2 (6)	C33—C32—C31	120.0 (6)
C8—C13—H13	119.9	C33—C32—H32	120.0
C12—C13—H13	119.9	C31—C32—H32	120.0
C10—C14—H14A	109.5	C34—C33—C32	120.5 (6)
C10—C14—H14B	109.5	C34—C33—H33	119.8
H14A—C14—H14B	109.5	C32—C33—H33	119.8
C10—C14—H14C	109.5	C33—C34—C29	118.6 (5)
H14A—C14—H14C	109.5	C33—C34—H34	120.7
H14B—C14—H14C	109.5	C29—C34—H34	120.7
C7—N1—S1	124.4 (4)	O9—C35—N3	122.2 (5)
C7—N1—H1N	125 (3)	O9—C35—C36	122.9 (5)
S1—N1—H1N	111 (3)	N3—C35—C36	114.9 (5)
O1—S1—O2	119.7 (2)	C37—C36—C41	120.1 (6)
O1—S1—N1	104.7 (2)	C37—C36—C35	121.5 (5)
O2—S1—N1	108.5 (2)	C41—C36—C35	118.4 (6)
O1—S1—C1	109.0 (2)	C36—C37—C38	120.8 (6)
O2—S1—C1	107.5 (2)	C36—C37—H37	119.6
N1—S1—C1	106.7 (2)	C38—C37—H37	119.6
C20—C15—C16	121.2 (6)	C39—C38—C37	117.3 (7)
C20—C15—S2	120.2 (5)	C39—C38—C42	122.0 (7)
C16—C15—S2	118.6 (5)	C37—C38—C42	120.8 (7)
C17—C16—C15	119.1 (6)	C40—C39—C38	122.7 (7)
C17—C16—H16	120.4	C40—C39—H39	118.7
C15—C16—H16	120.4	C38—C39—H39	118.7
C18—C17—C16	120.0 (7)	C39—C40—C41	119.7 (7)
C18—C17—H17	120.0	C39—C40—H40	120.2
C16—C17—H17	120.0	C41—C40—H40	120.2
C19—C18—C17	120.7 (7)	C40—C41—C36	119.6 (7)
C19—C18—H18	119.7	C40—C41—H41	120.2
C17—C18—H18	119.7	C36—C41—H41	120.2
C18—C19—C20	120.1 (7)	C38—C42—H42A	109.5
C18—C19—H19	119.9	C38—C42—H42B	109.5
C20—C19—H19	119.9	H42A—C42—H42B	109.5
C15—C20—C19	118.9 (6)	C38—C42—H42C	109.5
C15—C20—H20	120.6	H42A—C42—H42C	109.5
C19—C20—H20	120.6	H42B—C42—H42C	109.5
O6—C21—N2	121.4 (5)	C35—N3—S3	124.4 (4)
O6—C21—C22	122.7 (5)	C35—N3—H3N	123 (3)
N2—C21—C22	115.8 (4)	S3—N3—H3N	111 (3)
C27—C22—C23	119.0 (5)	O8—S3—O7	119.9 (3)
C27—C22—C21	117.0 (5)	O8—S3—N3	104.0 (2)
C23—C22—C21	124.1 (4)	O7—S3—N3	107.8 (2)
C22—C23—C24	121.2 (5)	O8—S3—C29	108.6 (2)
C22—C23—H23	119.4	O7—S3—C29	107.7 (2)
C24—C23—H23	119.4	N3—S3—C29	108.3 (2)
			
C6—C1—C2—C3	−0.4 (9)	C28—C24—C25—C26	−177.1 (6)
S1—C1—C2—C3	−179.4 (5)	C24—C25—C26—C27	−0.8 (11)
C1—C2—C3—C4	0.6 (11)	C25—C26—C27—C22	−1.1 (10)
C2—C3—C4—C5	−0.6 (12)	C23—C22—C27—C26	1.6 (9)
C3—C4—C5—C6	0.5 (12)	C21—C22—C27—C26	−178.5 (5)
C4—C5—C6—C1	−0.3 (10)	O6—C21—N2—S2	3.6 (7)
C2—C1—C6—C5	0.3 (9)	C22—C21—N2—S2	−175.3 (3)
S1—C1—C6—C5	179.3 (5)	C21—N2—S2—O5	−51.1 (5)
O3—C7—C8—C13	152.1 (6)	C21—N2—S2—O4	179.3 (4)
N1—C7—C8—C13	−28.9 (7)	C21—N2—S2—C15	64.8 (5)
O3—C7—C8—C9	−25.4 (8)	C20—C15—S2—O5	8.5 (6)
N1—C7—C8—C9	153.6 (5)	C16—C15—S2—O5	−170.1 (4)
C13—C8—C9—C10	0.3 (9)	C20—C15—S2—O4	142.5 (5)
C7—C8—C9—C10	177.9 (5)	C16—C15—S2—O4	−36.2 (5)
C8—C9—C10—C11	−1.9 (9)	C20—C15—S2—N2	−107.2 (5)
C8—C9—C10—C14	178.9 (6)	C16—C15—S2—N2	74.2 (5)
C9—C10—C11—C12	1.9 (10)	C34—C29—C30—C31	0.4 (8)
C14—C10—C11—C12	−178.9 (7)	S3—C29—C30—C31	−177.9 (4)
C10—C11—C12—C13	−0.3 (10)	C29—C30—C31—C32	−0.9 (8)
C9—C8—C13—C12	1.2 (8)	C30—C31—C32—C33	0.5 (10)
C7—C8—C13—C12	−176.3 (5)	C31—C32—C33—C34	0.4 (10)
C11—C12—C13—C8	−1.3 (9)	C32—C33—C34—C29	−0.9 (9)
O3—C7—N1—S1	−8.8 (8)	C30—C29—C34—C33	0.4 (8)
C8—C7—N1—S1	172.2 (4)	S3—C29—C34—C33	178.8 (5)
C7—N1—S1—O1	−173.1 (4)	O9—C35—C36—C37	147.1 (6)
C7—N1—S1—O2	−44.2 (5)	N3—C35—C36—C37	−32.8 (7)
C7—N1—S1—C1	71.4 (5)	O9—C35—C36—C41	−32.2 (8)
C2—C1—S1—O1	147.0 (5)	N3—C35—C36—C41	147.9 (5)
C6—C1—S1—O1	−31.9 (5)	C41—C36—C37—C38	−0.1 (9)
C2—C1—S1—O2	15.9 (5)	C35—C36—C37—C38	−179.5 (5)
C6—C1—S1—O2	−163.1 (4)	C36—C37—C38—C39	−0.3 (9)
C2—C1—S1—N1	−100.4 (5)	C36—C37—C38—C42	178.7 (6)
C6—C1—S1—N1	80.6 (5)	C37—C38—C39—C40	0.1 (11)
C20—C15—C16—C17	−0.7 (9)	C42—C38—C39—C40	−178.8 (7)
S2—C15—C16—C17	178.0 (5)	C38—C39—C40—C41	0.4 (12)
C15—C16—C17—C18	−0.2 (10)	C39—C40—C41—C36	−0.9 (11)
C16—C17—C18—C19	0.7 (12)	C37—C36—C41—C40	0.7 (9)
C17—C18—C19—C20	−0.4 (13)	C35—C36—C41—C40	−179.9 (5)
C16—C15—C20—C19	0.9 (10)	O9—C35—N3—S3	−6.2 (8)
S2—C15—C20—C19	−177.7 (5)	C36—C35—N3—S3	173.7 (4)
C18—C19—C20—C15	−0.4 (12)	C35—N3—S3—O8	179.9 (4)
O6—C21—C22—C27	−18.3 (7)	C35—N3—S3—O7	−51.8 (5)
N2—C21—C22—C27	160.5 (5)	C35—N3—S3—C29	64.5 (5)
O6—C21—C22—C23	161.6 (5)	C30—C29—S3—O8	144.2 (4)
N2—C21—C22—C23	−19.6 (7)	C34—C29—S3—O8	−34.2 (5)
C27—C22—C23—C24	−0.3 (8)	C30—C29—S3—O7	13.0 (5)
C21—C22—C23—C24	179.9 (5)	C34—C29—S3—O7	−165.4 (4)
C22—C23—C24—C25	−1.5 (8)	C30—C29—S3—N3	−103.4 (4)
C22—C23—C24—C28	177.7 (5)	C34—C29—S3—N3	78.2 (5)
C23—C24—C25—C26	2.1 (9)		

### Hydrogen-bond geometry (Å, º)

**Table:**

*D*—H···*A*	*D*—H	H···*A*	*D*···*A*	*D*—H···*A*
N1—H1*N*···O7^i^	0.85 (2)	2.04 (2)	2.864 (5)	164 (5)
N2—H2*N*···O2^ii^	0.86 (2)	2.15 (3)	2.920 (5)	150 (5)
N3—H3*N*···O6	0.85 (2)	2.05 (2)	2.891 (5)	172 (5)

Symmetry codes: (i) *x*−1, *y*, *z*+1; (ii) *x*, *y*, *z*−1.
